# Impact on life expectancy of late diagnosis and treatment of HIV-1 infected individuals: UK CHIC

**DOI:** 10.1186/1758-2652-13-S4-O27

**Published:** 2010-11-08

**Authors:** MT May, M Gompels, CA Sabin

**Affiliations:** 1 Bristol University, Social and Community Medicine, Bristol, UK; 2 North Bristol NHS Trust, Bristol, UK; 3 UCL, Medical School, London, UK

## Purpose of the study

Life expectancy (LE) is an important health indicator that informs decisions both by individuals and public policymakers. The pattern of the HIV epidemic and LE varies by country. Previous estimates of LE in HIV-infected populations have included few UK patients[1]. We assess time trends, sex-differences and the impact of delayed treatment on LE in treated HIV patients in the UK and compare with the LE of the UK population.

## Methods

We analysed data from the UK CHIC cohort study on patients aged over 20 years who started cART in 1996-2008 with CD4≤ 350cells/mm^2^ (excluding injection drug users). All-cause mortality was ascertained from clinic records and by linkage to the death registry. Abridged life tables were constructed from age-specific mortality rates (5year age bands) to estimate LE for ages 20-65 years. Results are presented as LE at exact age 20, the average additional years that will be lived by a person after age 20, according to the cross-sectional age-specific mortality rates during the study period. We estimated LE overall and by period (1996-99, 2000-02, 2003-05, 2006-08). We compared LE in those treated for HIV with the UK population stratified by sex. To assess the impact of late treatment, we estimated LE stratified by CD4 cell count at start of cART in those treated post 2000 who were ART-naive.

## Results

1248/17661 eligible patients died during 91203 pyrs fup. 75% of patients were male, 58% white. Transmission risk group was 54% MSM, 37% heterosexual and 9% unknown. At start of cART, median age (IQR) was 37(32-43) years, median CD4 cell count 166 (75-241) and the proportion with no ART exposure prior to cART increased from 54% in 1996-99 to 96% in 2006-08. LE (standard errors) at exact age 20 years increased from 30.0 (1.2) to 45.8 (1.7) years from 1996-99 to 2006-08. Over the study period LE for male patients was 39.5(0.45) and for female 50.2(0.45) years compared with 57.8 and 61.6 years for men and women in the UK population (1996-2006). LE was 37.9 (1.3), 41.0 (2.2) and 53.4 (1.2) years in those starting cART with CD4<100, 100-199 and 200-350 cells/mm^2^ respectively (N=9657).

## Conclusions

LE of those treated for HIV-infection in the UK has increased by over 15 years during 1996-2008, but remains about 13 years less than that of the UK population. The higher LE of women compared with men is magnified in the HIV-infected population. Earlier diagnosis through improved screening and timely treatment might increase LE.
